# Annular Pancreas Causing Gastric Diverticulum in a 29-Year-Old Man: A Rare Complication of an Uncommon Condition

**DOI:** 10.14309/crj.0000000000001667

**Published:** 2025-04-07

**Authors:** Sarpong Boateng, Gabriel Heymann, Frances Mejia, Benjamin A. Lerner, Caroline Loeser

**Affiliations:** 1 Department of Medicine, Bridgeport Hospital, Bridgeport, CT; 2 Department of Medicine, Section of Gastroenterology, Bridgeport Hospital, Bridgeport, CT; 3 Department of Medicine, Yale School of Medicine, New Haven, CT

## CASE REPORT

Annular pancreas (AP) is a rare congenital anomaly characterized by pancreatic tissue encircling the duodenum, leading to variable degrees of obstruction.^1,2^ In adults, AP is often misdiagnosed because of its nonspecific presentation.^3^ Chronic duodenal obstruction may result in complications, including gastric diverticulum.^4^

**Figure 1.**
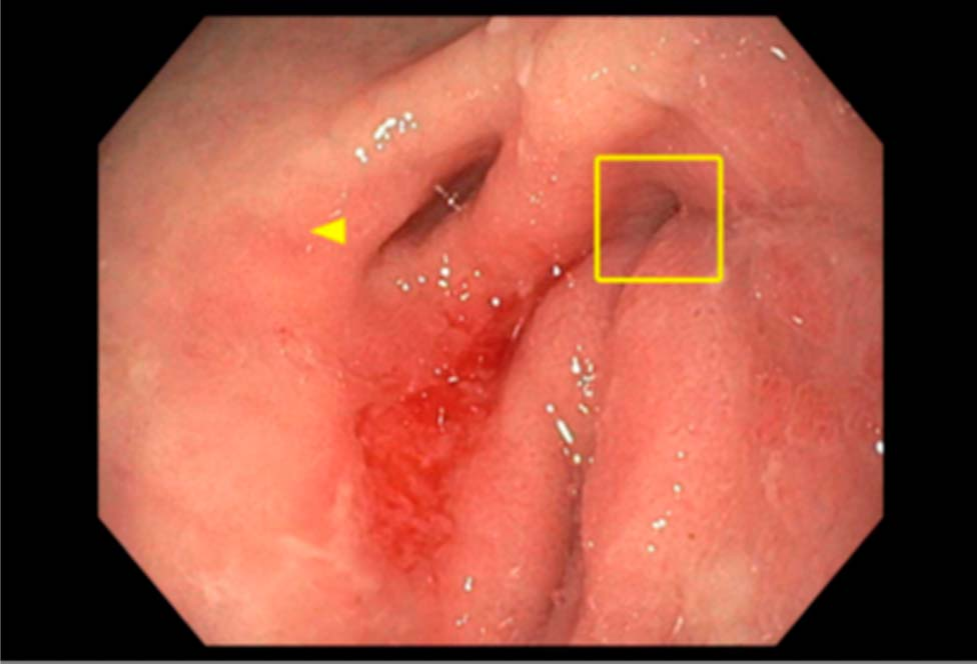
Upper endoscopy showing prepyloric diverticulum of stomach (yellow arrow) and pylorus (box).

**Figure 2.**
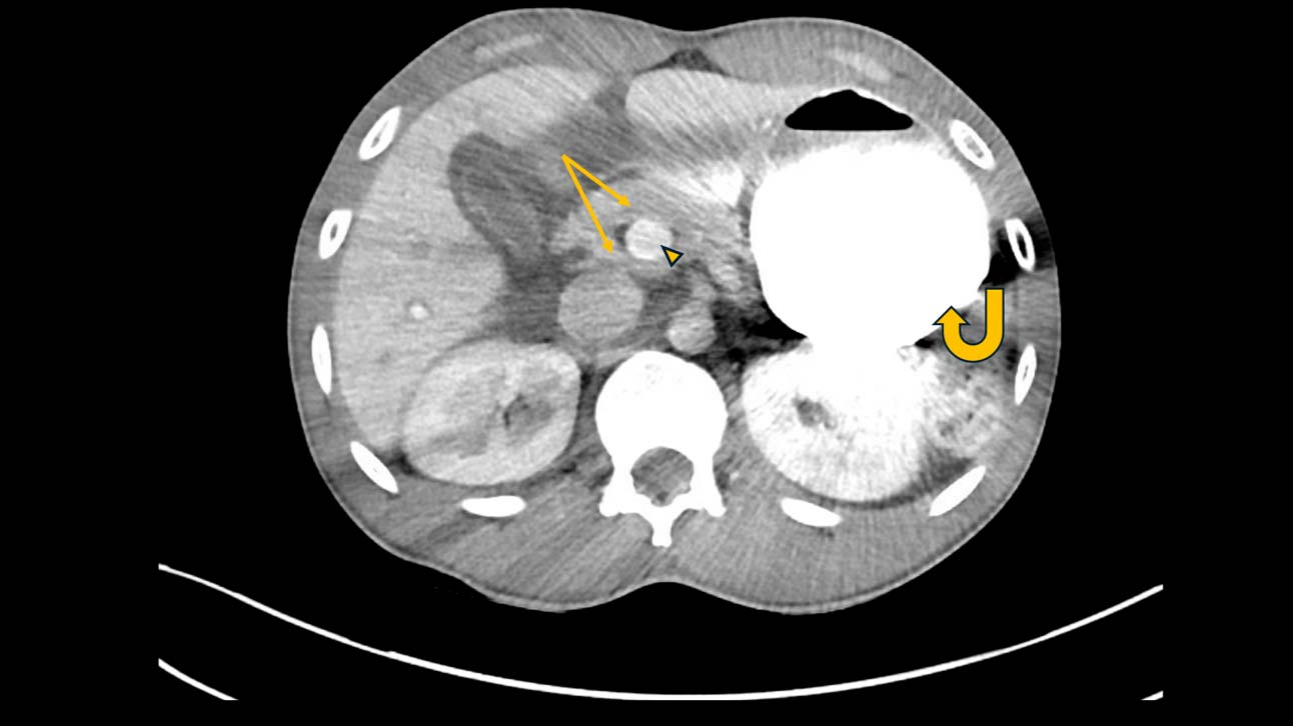
Contrast-enhanced axial abdominal computed tomography scan highly suggestive of annular pancreas configuration (arrows), encircling the second portion of the duodenum (arrowhead), and a distended stomach (curved arrow).

**Figure 3.**
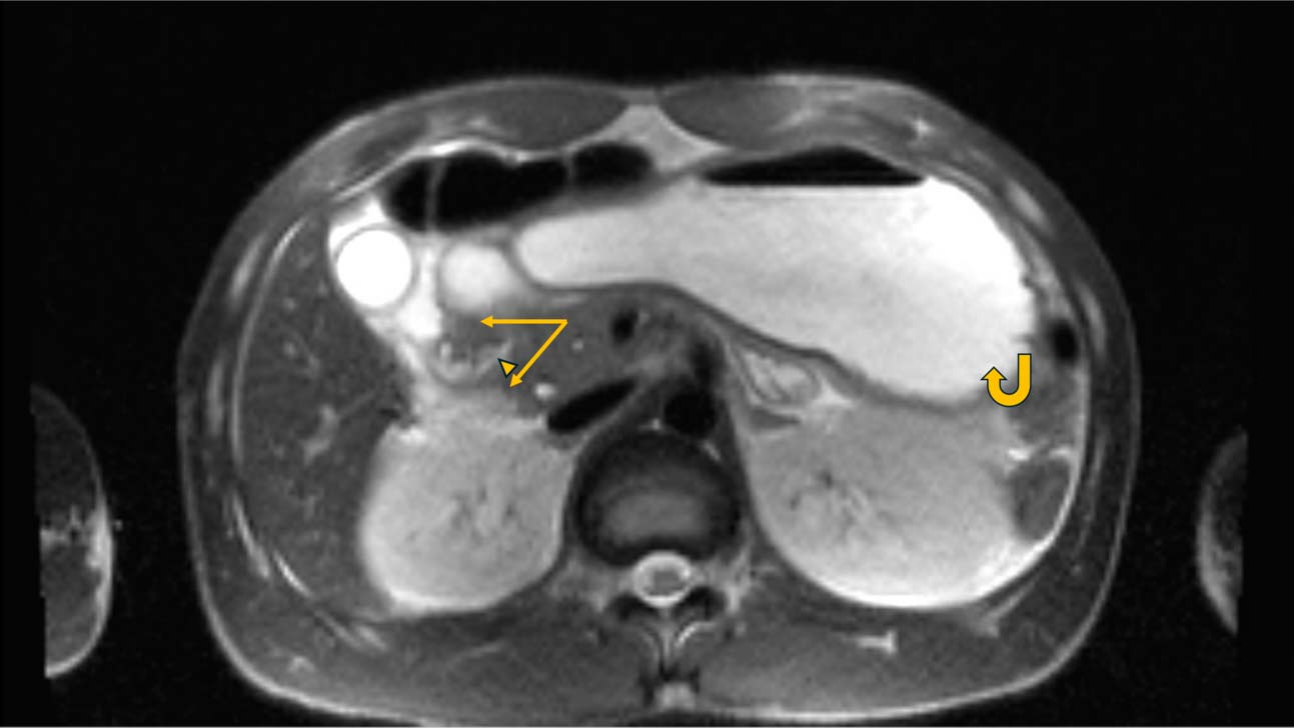
Axial T1 magnetic resonance imaging depicting a complete annular pancreas (arrows) around the proximal second portion of the duodenum(arrowhead), with an enlarged stomach (curved arrow).

A 29-year-old man presented with a 5-year history of recurrent postprandial abdominal pain, early satiety, and nonbilious vomiting. He reported progressive weight loss over the previous year. Prior evaluations, including a noncontrast computed tomography in his home country (Haiti), were unremarkable. A repeat evaluation in the United States revealed persistent gastric distention. Upper endoscopy revealed retained food and a prepyloric diverticulum (Figure 1). A 5.8-mm ultra-slim endoscope failed to pass through the pylorus. Contrast-enhanced computed tomography and magnetic resonance imaging demonstrated a complete AP encircling the second portion of the duodenum with upstream gastric dilation (Figures 2 and 3). The patient underwent laparoscopic gastrojejunostomy, which confirmed duodenal obstruction due to AP. Given the limited space, a hand-sewn gastrojejunostomy was performed. Postoperatively, the patient recovered well, tolerating a full oral diet. Follow-up showed complete symptom resolution.

This case highlights AP as a rare but important consideration in adults with unexplained gastric outlet obstruction and the potential for secondary gastric diverticulum formation.

## DISCLOSURES

Author contributions: S. Boateng: Conceptualization, manuscript drafting, literature review, final approval and is the article guarantor. G. Heymann: Critical revision, imaging interpretation, final manuscript approval. F. Mejia: Case review, manuscript editing, final approval. BA Lerner: Surgical insights, manuscript review, final approval. C. Loeser: Supervision, manuscript review, final approval.

Financial disclosure: None to report.

Informed consent was obtained for this case report.
